# Lactating mothers' perceptions and sensory acceptability of a provitamin A carotenoid–iron‐rich composite dish prepared from iron‐biofortified common bean and orange‐fleshed sweet potato in rural western Uganda

**DOI:** 10.1002/fsn3.4053

**Published:** 2024-03-08

**Authors:** Edward Buzigi, Kirthee Pillay, Muthulisi Siwela, Blessing Mkhwanazi, Mjabuliseni Ngidi, Joshua Ssozi, Babra Muhindo Mahinda, Evyline Barugahara Isingoma

**Affiliations:** ^1^ Department of Community Health & Behavioural Sciences School of Public Health, College of Health Sciences, Makerere University Kampala Uganda; ^2^ Department of Dietetics and Human Nutrition School of Agricultural, Earth and Environmental Sciences, University of KwaZulu‐Natal Pietermaritzburg South Africa; ^3^ Department of Public Health & Nutrition Faculty of Health Sciences Victoria University Kampala Uganda; ^4^ School of Vocational Studies, Kyambogo University Kampala Uganda

**Keywords:** iron‐biofortified common bean, lactating mothers, non‐iron‐biofortified common bean, orange‐fleshed sweet potato, perceptions, sensory acceptability, white‐fleshed sweet potato

## Abstract

Uganda's lactating mothers are vulnerable to deficiencies of vitamin A and iron because they consume plant‐based conventional foods such as white‐fleshed sweet potato (WFSP) and non‐iron biofortified common bean (NIBCB) that are low in provitamin A (PVA) and iron, respectively. A PVA carotenoid–iron‐rich dish was prepared from a combination of orange‐fleshed sweet potato (OFSP) and iron‐biofortified common bean (IBCB). This study evaluated the perceptions and sensory acceptability of OFSP+IBCB (test food) against WFSP+NIBCB (control food) among lactating mothers in rural Uganda. A total of 94 lactating mothers participated in the study. The sensory attributes (taste, color, aroma, texture, and general acceptability) of test and control foods were rated using a five‐point facial hedonic scale (1 = dislike very much, 2 = dislike, 3 = neutral, 4 = like 5 = like very much). An attribute was acceptable if the participant scored from like to like very much. Focus group discussions (FGDs) were conducted to assess participant perceptions about their future consumption of OFSP+IBCB. The chi‐square test was used to detect the proportion difference for each sensory attribute between OFSP+IBCB and WFSP+NIBCB, while FGD data were analyzed by thematic analysis. Taste, color, and aroma were acceptable to the mothers and not significantly different between OFSP+IBCB and WFSP+NIBCB (*p* > .05). Participants had positive perceptions of the taste, aroma, and color of the OFSP+IBCB and negative perceptions about the soft texture of OFSP. The lactating mothers had positive perceptions of consuming OFSP+IBCB provided they were accessible, affordable, and feasible to prepare.

## Graphical abstract

A provitamin A carotenoid–iron‐rich composite dish prepared from iron‐biofortified common bean and orange‐fleshed sweet potato is acceptable among lactating mothers in rural Uganda. A figure that best represents the scope of the paper. The image supplied should fit within the dimensions of 50 mm x 60 mm, and be fully legible at this size.

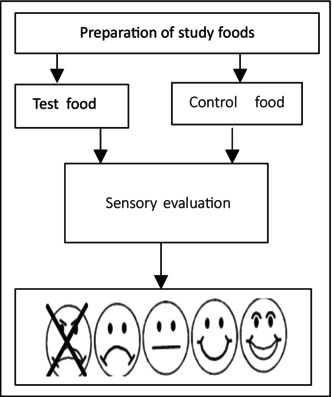

## 1 INTRODUCTION

Dietary iron deficiency (ID) and vitamin A deficiency (VAD) are the two leading micronutrient deficiencies of public health nutrition importance that negatively impact women of reproductive age (WRA) residing in low‐ and middle‐income countries (LMICs) of Africa and Asia (Abbafati et al., 2020; Gardner & Kassebaum, 2020; Mawani & Aziz Ali, 2016). However, the highest burden is among pregnant and lactating women because of the increased physiological demands for iron and vitamin A, which increase due to either fetal demands during pregnancy or breastfeeding during lactation (Gannon et al., 2020; Khayat et al., 2017). If vitamin A requirements during lactation are not met, it may be associated with clinical VAD characterized by night blindness and bitot's spots among lactating mothers (Baytekus et al., 2019). The increased iron demands among WRA coupled with the intake of low‐iron foods are associated with ID anemia, the leading cause of maternal anemia in LMICs (Chaparro & Suchdev, 2019). Moreover, the progress on anemia in WRA (15–49 years) is insufficient to meet the World Health Assembly's global nutrition target to reduce anemia prevalence by 50% by the year 2030 in LMICs, including Uganda (Stevens et al., 2022).

The recommended dietary allowance (RDA) is the average daily dietary intake level that is sufficient to meet the nutrient requirements of nearly all healthy individuals in a particular life stage and sex group. It is worth noting that the RDA for vitamin A among lactating mothers is higher at 885–1300 μgRAE/day compared to pregnant women at 700 μgRAE/day (Gannon et al., 2020; Kominiarek & Rajan, 2016). Moreover, the RDA for iron needed by lactating mothers is high at 10–30 mg/day compared to 8 mg/day for adult males (Ross et al., 2014; World Health Organization, 2016). To contribute toward achieving the RDA for iron among lactating mothers, iron supplementation programs are recommended by the World Health Organization (WHO) during the postpartum period, beginning just after neonate delivery to the first 6 weeks after delivery (World Health Organization, 2016). In contrast, the WHO does not recommend vitamin A supplementation during postpartum because it has neither health benefits to the mother nor the infant (World Health Organization, 2011). However, it recommends that the lactating mother should consume a diversified diet including vitamin A‐rich foods (World Health Organization, 2011). It is worth noting that vitamin A and iron supplementation during postpartum have the potential to improve the breast milk content of vitamin A and iron, respectively (Holm et al., 2016; Oliveira et al., 2016). This is important because it improves the vitamin A intake of the breastfeeding child, and therefore may improve the vitamin A and iron status of the breastfeeding infant. However, postpartum vitamin A and iron supplementation programs are not sustainable because they are done for only the first 6 weeks after delivery, yet lactation should go up to 2 years and beyond as recommended by the WHO (World Health Organization & United Nations Children's Fund, 2021). Therefore, a continued intake of either vitamin A and iron‐fortified foods or vitamin A and iron animal source foods such as meat and liver would be necessary to improve the vitamin A and iron status of lactating mothers and their breast milk (Mwangi et al., 2021). However, vitamin A and iron‐fortified foods are either expensive or not available on the market to be accessed by poor mothers who reside in LMICs and rural settings.

A high proportion of lactating mothers residing in rural Uganda are at an increased risk of developing VAD and ID anemia because they habitually consume staple plant‐source foods (PSFs) that are low in PVA carotenoids and iron (Nankumbi et al., 2023). The staple PSFs are usually either tubers such as conventional non‐PVA biofortified sweet potato (*Ipomoea batatas*), white‐fleshed sweet potato (WFSP), or low‐iron conventional non‐iron biofortified common bean (NIBCB) such as *Nambale*. Plant biofortification is a nutrition‐sensitive strategy that increases the concentration of target nutrients, particularly micronutrients, such as iron and vitamin A in the edible portions of staple food crops through conventional breeding, fertilizer application, or bioengineering (recombinant DNA technology) to improve the nutritional status of target consumers (Vasconcelos et al., 2017). Compared to other nutrition‐specific strategies such as industrial fortification and supplementation traditionally used to combat VAD and ID, biofortification is relatively cost‐effective because once biofortified seeds are released, the rural poor from LMICs can plant them season after season for sustained consumption (Bouis & Saltzman, 2017; Glahn & Noh, 2021).

To contribute toward improved vitamin A and iron intake among vulnerable groups such as lactating mothers, the government of Uganda through HarvestPlus introduced PVA biofortification and iron biofortification programs for its staple foods such as sweet potato (*Ipomoea batatas*) and common bean (*Phaseolus vulgaris)*, respectively (Atero, 2019; HarvestPlus, 2012; Hotz et al., 2012). Therefore, the PVA‐biofortified sweet potato, orange‐fleshed sweet potato (OFSP), and iron‐biofortified common bean (IBCB) were introduced in Uganda. Introducing iron‐biofortified foods and provitamin A‐biofortified foods is increasingly seen as a potential strategy for combating ID and VAD in LMICs of Africa (Finkelstein et al., 2017; Govender et al., 2019b; Gurmu et al., 2014). It is worth noting that understanding the acceptability of these biofortified foods is a prerequisite for future consumer utilization of such biofortified foods (Huey et al., 2023; Siwela et al., 2020).

In Uganda, a dish prepared from a combination of WFSP and NIBCB is commonly consumed among WRA including lactating mothers (Uganda Bureau of Statistics & Inner City Fund, 2018). However, a combination of WFSP and NIBCB is low in PVA carotenoids and iron, respectively (Drapal & Fraser, 2019). When such a combination is habitually eaten, it has the potential to increase the risk of VAD and ID among Ugandan consumers (Kyamuhangire et al., 2013; Tidemann‐Andersen et al., 2011). To contribute toward improving PVA carotenoid and iron intake among lactating mothers in rural Uganda, a PVA carotenoid–iron‐rich innovative dish was prepared from a combination of OFSP and IBCB released in Uganda. This is plausible because the former is rich in PVA carotenoids (Neela & Fanta, 2019), and the latter is rich in iron (Luna et al., 2020). Therefore, this study evaluated the perceptions and sensory acceptability of a PVA carotenoid–iron‐rich OFSP+IBCB (test composite foods) against the low PVA carotenoid–iron WFSP+NIBCB (control composite foods) among lactating mothers in rural Uganda. Findings generated from this study inform whether a dish of OFSP+IBCB rich in PVA carotenoids and iron has the potential to replace the conventional dish WFSP+NIBCB, which is low in PVA carotenoids and iron among Ugandan lactating mothers.

## 2 MATERIALS AND METHODS

### 2.1 Study setting and design

This experimental cross‐over sensory acceptability study was conducted in Bwera General Hospital, located in Kasese district, western Uganda. The study was conducted in Kasese district because according to the most recent Uganda Demographic and Health Survey, Kasese is among the districts located in the Tooro region (Western Uganda) with the highest prevalence (over 50%) of anemia among WRA (Uganda Bureau of Statistics & Inner City Fund, 2017). Bwera Hospital was specifically chosen because it is a district referral hospital that receives over 500 lactating mothers in a month for postnatal services from all over the district.

### 2.2 Study participants and sample size determination

The lactating mothers rated the taste, texture, aroma, color, and overall acceptability of both the taste food and control samples using a five‐point facial hedonic scale (1 = dislike very much = 1, dislike = 2, neutral, 3, like = 4, and like very much = 5). A participant was considered to accept the composite food if she scored 4 and above (like to like very much). Therefore, the sample size was determined to test the hypothesis that the mean score of the sensory attribute would be 4 and above. The sample size was calculated by use of the statistical considerations for a cross‐over study software available at http://hedwig.mgh.harvard.edu/sample_size/js/js_crossover_quant.html with the following parameters and assumptions. The probability is 80% that the study will detect a treatment difference at a two‐sided .05 significance level if the true difference between the study foods is a sensory acceptability score of 0.02. This was based on the assumption that the within‐participant standard deviation of the sensory acceptability variable is 0.02. To this end, a sample size of 65 lactating mothers for each study composite dish would therefore allow us to reject the null hypothesis with 80% power. Moreover, a minimum sample size of 60 participants is considered adequate for a valid sensory acceptability study (Hough et al., 2006).

### 2.3 Sampling procedure, inclusion and exclusion criteria of study participants

The lactating mothers were recruited by systematic sampling method. In systematic sampling, a number is assigned to every record, and then every nth record is selected from a list (Stewart, 2016). On average, 30 lactating mothers come for postnatal services in Bwera General Hospital every day. This study systematically recruited 10 of 30 lactating mothers per day in the postnatal clinic of Bwera General Hospital between the 4th and 15th of August 2023. The following procedure was followed: Every morning, a list of all the first 30 lactating mothers to come for postnatal services was obtained. To this end, 30/10 = 3. Therefore, every third lactating mother was recruited. A number from 1 to 3 was chosen at random as a starting point. In this case, number 3 was chosen at random. Therefore, every third lactating mother was recruited in the study. That is to say, lactating mothers numbered 3, 6, 9, 12, 15, 18, 21, 24, 27, and 30 were recruited every day until the calculated sample size was reached. A study participant was included in the study if she had a breastfeeding child. A lactating mother was excluded from the study if she had any form of illness. This exclusion criterion was considered because several illnesses may affect sensory variables such as smell and taste in mothers during the period of lactation (Cingi et al., 2022).

### 2.4 Pilot study

A pilot study is a preliminary step of the entire research protocol and is often a smaller scale investigation that aids in the preparation and adjustment of the main study. The pilot study was conducted to test the feasibility of methods and procedures for the preparation of study foods, sensory evaluation and focus group discussions (FGDs) for later use in the main study. The village health team members (community health workers) identified five peer mothers from the community to participate in the preparation and cooking of the study foods. The peer mothers advised on the common cooking methods used to prepare common beans and sweet potatoes at the household level. For example, they advised that common bean is soaked in water overnight (approximately 8–10 h) before it is boiled and fried for consumption, while sweet potato is peeled and cooked by local steaming methods. Moreover, boiling common bean with prior soaking and steaming OFSP have demonstrated to have a higher retention of iron and PVA carotenoids, respectively (Buzigi et al., 2020c; Yao et al., 2023). These cooking methods were adopted in the preparation of study composite dishes for sensory evaluation during the main study. The midwives identified 20 lactating mothers from the postnatal clinic in Bwera Hospital to participate in the sensory evaluation and FGD pilot study.

A total number of 20 lactating mothers participated in the sensory evaluation pilot study. A short while later, all the 20 lactating mothers who had participated in the sensory evaluation participated in the pilot FGDs. Two FGDs were conducted during the pilot study and each focus group had 10 lactating mothers. The pilot study was conducted 1 week before the main study. This pilot study was conducted on a different day from the main study to prevent the pilot study participants from participating in the main study. The pilot study established that the majority (98%) of the lactating mothers who participated in the pilot study had low literacy levels (did not complete primary education). This was confirmed by records from the postnatal clinic. Knowing the literacy level of the potential study participants guided the main study to modify the sensory evaluation hedonic scale from a seven‐point scale to a five‐point scale. It is worth noting that longer hedonic scales, such as those with 7 or 9 rating scales, tend to confuse participants with lower literacy levels, while rating scales that are shorter than the five‐point scale tend to cause end point avoidance (Stone & Sidel, 2004). As the pilot study venue was too far away from the postnatal clinic, a closer alternative venue was identified for the main study. No changes were made to the FGD guide after the pilot study. The pilot study was also used to train research assistants. This helped the research assistants to get acquainted with the implementation of the sensory evaluation questionnaire and focus group discussion guide.

### 2.5 Description of the control and test composite dishes used in the study

#### 2.5.1 Ingredients for the preparation of the study composite dishes

**FIGURE 1:**
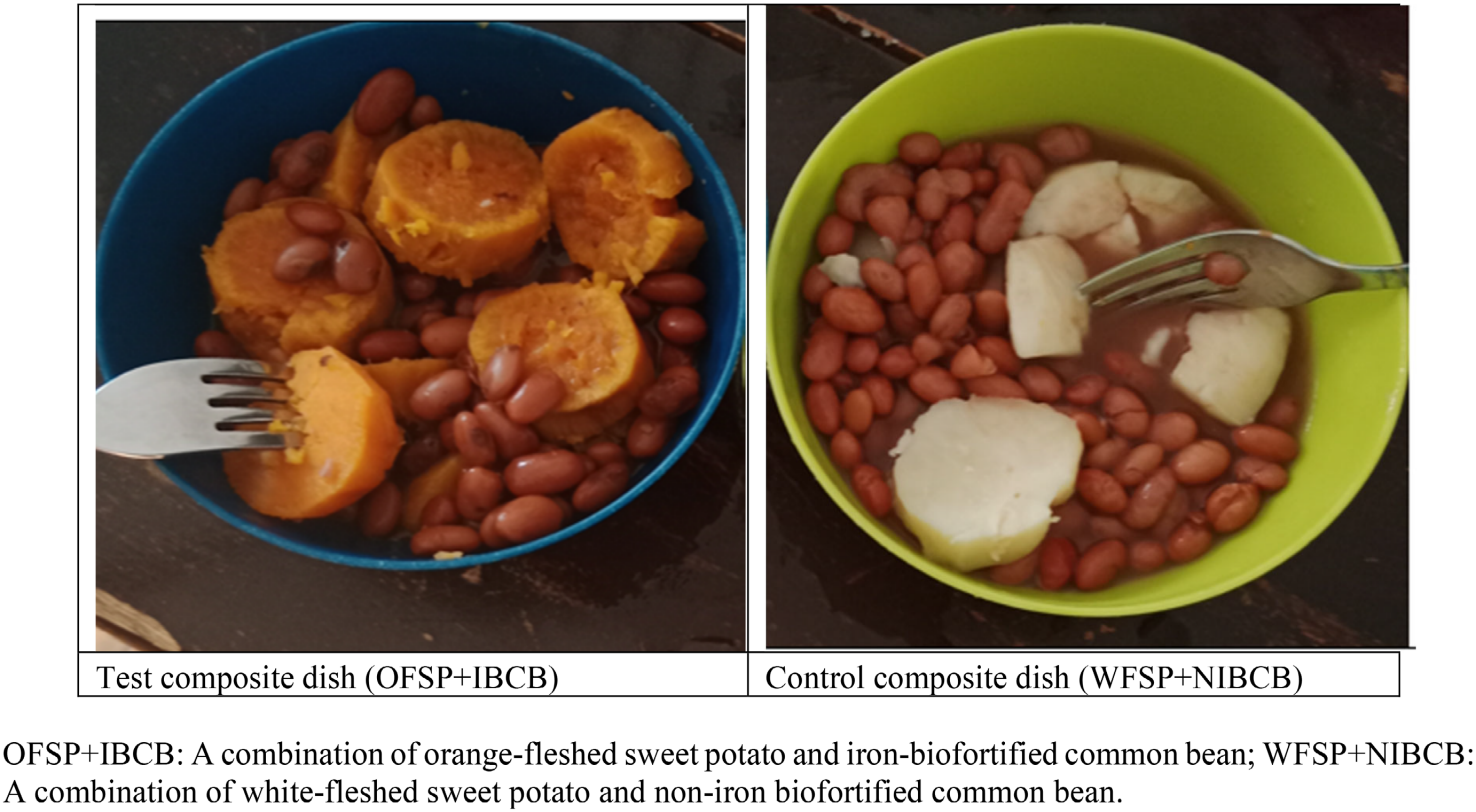
Test and control composite dishes prepared from sweet potato and common bean.

Composite foods are characterized by being multi‐ingredient and include both ready‐to‐eat products and home‐prepared dishes (Durazzo et al., 2020). This study used home‐based cooking methods commonly used to prepare common beans and sweet potatoes in Uganda. The control composite dish was WFSP served with NIBCB (WFSP+NIBCB). This combination was selected because it is a non‐biofortified dish habitually consumed in Uganda and it is characterized by low PVA carotenoids and iron. The innovative test composite dish was OFSP served with IBCB (OFSP+IBCB). The test composite dish was selected because the former is biofortified with PVA carotenoids, while the latter is biofortified with iron. Figure 1 shows the ingredients used to prepare the control and test composite dishes for the acceptability study.

#### 2.5.2 Preparation of test and control composite dishes

The village health team members (community health workers) identified five expert peer mothers from the local community and invited them to Bwera Hospital kitchen to participate in the preparation of the study composite dishes. Expert peer mothers were encouraged to prepare the composite dishes using locally acceptable home‐based methods used in the community to prepare common beans and sweet potatoes to be consumed by lactating mothers. The biofortified foods, OFSP (*NKB 135*) and IBCB (*NARO bean 4c*) were procured from the National Crops Resources Research Institute (NaCRRI), Namulonge, Uganda (National Crops Resources Research Institute, 2023). The commonly consumed WFSP (*Ebiribwa*) and NIBCB (*Nambale*) in the community were procured from the local market by expert peer mothers who prepared the composite dishes used in the study in the presence of the research assistants. The peer mothers soaked the NIBCB and IBCB separately overnight (approximately 8–10 h) before cooking them by boiling. The mothers chose to boil common beans with prior soaking because they argued that soaking softens common beans to reduce cooking time and saves fuel as reported in a previous Ugandan study (Buzigi et al., 2020c). The IBCB and NIBCB were cooked separately using a local stove (*Esigiri*) until they were soft and ready for human consumption. The cooking time for NIBCB and IBCB was approximately 1.5 and 3.5 h, respectively. Expert peer mothers prepared OFSP and WFSP separately by peeling, and washing, followed by steaming. The local method of steaming involved putting 1 liter of tap water into the cooking pot, followed by banana stalks, then banana leaves, in which sweet potato was wrapped before being put onto the hot charcoal stove for steaming. The role of the banana stalk was to create an elevation and separation between the water in the cooking pot from the banana leaves, where the sweet potatoes were wrapped. To this end, when water in the pot boiled, it released steam that vaporized through the banana stalk spaces to heat the banana leaves in which the sweet potatoes were wrapped. The cooking time for WFSP and OFSP was approximately 45 and 35 min, respectively. The duration of boiling and steaming was sufficient to soften the plant tissue as assessed by the penetration of the tip of a knife without resistance (Buzigi et al., 2020c).

#### 2.5.3 Provitamin A carotenoids, vitamin A, and iron composition of cooked study foods

The PVA carotenoid composition of cooked sweet potato was analyzed by high‐performance liquid chromatography (HPLC) as described in the HarvestPlus handbook for carotenoid analysis (Rodriguez‐Amaya & Kimura, 2004). The vitamin A composition was calculated by using the Institute of Medicine (2001) bioconversion rates of PVA carotenoids to vitamin A (retinol activity equivalents), that is, 12 μg of β‐carotene is equivalent to 1 μg of retinol, while 24 μg of α‐carotene is equivalent to 1 μg retinol (Institute of Medicine, 2001). The iron composition of the common bean was analyzed by flame atomic absorption spectroscopy as described elsewhere (Buzigi et al., 2020c). Triplicate analysis for each variety of cooked sweet potato and common bean was conducted to get an average of each micronutrient in each food. Table 1 shows the PVA carotenoid and iron composition of the cooked sweet potato and common bean, respectively, used in the study.

**TABLE 1:** Provitamin A carotenoid (retinol activity equivalent) and iron composition of study cooked sweet potato and common bean, respectively, used in the study.

Micronutrient	OFSP/100 g	WFSP/100 g	IBCB/100 g	NIBCB/100 g
β‐carotene (μg)	36,900	9200	Not tested	Not tested
α‐carotene (μg)	300	110	Not tested	Not tested
Vitamin A (μgRAE)	3088	711	Not tested	Not tested
Iron (mg)	Not tested	Not tested	9.3	5.1

*Note*: The values are presented on a cooked (fresh) basis.

Abbreviations: IBCB, iron‐biofortified common bean; NIBCB, non‐iron‐biofortified common bean; OFSP, orange‐fleshed sweet potato; RAE, retinol activity equivalent (retinol); RAE, β‐carotene (μg/100 g)/12+ α‐carotene (μg/100 g)/24 (Institute of Medicine, 2001); WFSP, white‐fleshed sweet potato.

### 2.6 Measurement of study variables

#### 2.6.1 Measurement of sensory acceptability

**FIGURE 2:**
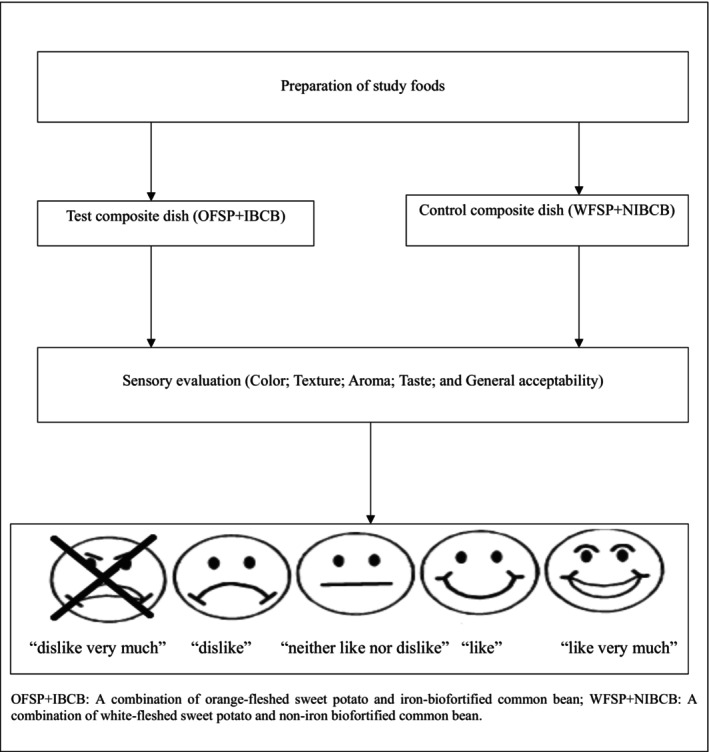
Flow of sensory acceptability of study composite dishes.

Sensory acceptability was measured by the sensory evaluation method. The sensory evaluation sessions were held in a room with separate, isolated cubicles set up for each panelist. Each panelist received a spoon and a small polystyrene cup containing the control composite dish, approximately 50 g of NIBCB combined with 50 g WFSP (WFSP+NIBCB), and the test composite dish, approximately 50 g of IBCB combined with 50 g of OFSP (OFSP+IBCB). To prevent lactating mothers from making judgments based on labels, the samples were given random numbers as labels. The purpose of this was to ensure that panelists relied solely on their sensory experiences to assess the samples (Lawless & Heymann, 2010). To achieve this, the samples were randomly labeled with a unique three‐digit code obtained from a table of random numbers and were served in a random sequence (Lawless & Heymann, 2010). The samples were warmed in a microwave oven for 10 seconds on medium heat, before serving. The lactating mothers were provided with a cup of water to rinse their palates between evaluating samples. Before each session, the sensory attributes of color, texture, aroma, taste, and overall acceptance were explained to the panelists (lactating mothers). An equally spaced five‐point facial hedonic scale with ratings (1=“dislike very much”; 2 = “dislike”; 3=“neither like nor dislike”; 4 = “like”; 5= “like very much”) was used. It is worth noting that the WRA in the study district have lower literacy levels, with 67% of children dropping out of school before completing ordinary‐level education (National Population Council, 2021). This coupled with low literacy levels established during the pilot study justifies the use of the five‐point rating scale in the study participants as recommended by Stone and Sidel (2004). Each sensory attribute was described on the form with an accompanying facial hedonic scale. The ratings of the hedonic scale were verbally explained to the panelists in the local language during the sensory evaluation sessions. The participants were asked to rate the acceptance of each attribute by marking the appropriate response on the facial hedonic scale. A sensory attribute was considered acceptable if it was rated as “like to like very much.” Figure 2 shows the flow of sensory acceptability of the study composite dishes.

#### 2.6.2 Measurement of perceptions

Qualitative data (perceptions) were collected using FGDs and a FGD guide. The FGDs were conducted to explore the lactating mothers' perceptions to understand the factors that may motivate them to consume the test composite dish (OFSP+IBCB). The FGDs were conducted about 30 min after the sensory evaluation study was completed. All the 94 lactating mothers who participated in the sensory evaluation study were eligible to participate in the FGDs. Through established community relationships, four facilitators, experienced in conducting FGDs in the local language (Lhukonzo), were recruited for 2 days of training in focus group discussion moderation concerning the specifics of this research study following guidelines explained elsewhere (Amico et al., 2011). A trained facilitator directed the discussions, using an unstructured FGD guide. The FGD guide consisted of a brief explanation of the samples that were tasted during the sensory evaluation as well as the question for initiating and facilitating the discussion. Guidelines for conducting FGDs with a structured set of open‐ended questions were followed, as recommended elsewhere (Krueger & Casey, 2015). The open‐ended question was “You have participated in the sensory evaluation of OFSP combined with IBCB. What would motivate you to consume the IBCB+OFSP composite dish?” Furthermore, the facilitators used probes such as “Would you explain further?” and “Would you give an example?” where it was deemed necessary. The FGDs were facilitated in the local language (Lhukonzo) by trained FGD facilitators. A digital voice recorder was used to record the FGDs after participants consented to the use of the voice recorder. A sample size for an FGD between 7 and 12 participants is appropriate for a qualitative nutrition‐related study (Harris et al., 2009). Therefore, each focus group included 8–10 participants. The FGDs were conducted until data saturation was reached. Data saturation means that researchers are not finding any additional or new data from the FGDs (Hennink & Kaiser, 2022).

### 2.7 Data analysis

#### 2.7.1 Analysis of quantitative data generated from sensory acceptability measurements

The proportion (percentage) of lactating mothers was calculated according to their sensory attribute ratings for the control composite dish (WFSP+NIBCB) and test composite dish (OFSP+IBCB). A sensory attribute was considered acceptable if it was rated as good to very good by the lactating mothers. A binary outcome of yes/no was created for sensory acceptability. A chi‐square statistical test was used to test for significant differences in the sensory attributes (taste, color, aroma, texture, and general acceptability) between the control and test composite dishes. A chi‐square test was considered significant at a *p* < .05. Statistical data analysis was done using STATA, version 15.1.

#### 2.7.2 Analysis of qualitative data generated from focus group discussions

Qualitative data generated from FGDs were analyzed using thematic analysis by following the six steps including familiarization of data, coding of data, generating of themes, reviewing of themes, defining and naming of themes, and writeup (Braun & Clarke, 2006). Thematic analysis is a method for identifying, analyzing, and reporting patterns (themes) within data. Instead of starting with predetermined themes based on theory or existing knowledge (deductive thematic analysis), this study used an inductive thematic analysis approach, where the themes were determined by the data itself. Table 2 shows the description of the six steps used during the thematic analysis of data generated from the FGDs as recommended by Braun and Clarke (Braun & Clarke, 2006).

**TABLE 2:** Description of the six steps for inductive thematic analysis used in the study (Braun & Clarke, 2006).

Steps	Description
Familiziation with data	A verbatim transcription was done. Verbatim transcription is a word‐for‐word transcription of a recording. The verbatim transcription was later translated from the local language (*Lhukonzo*) into English by the three FGD facilitators. The translated recordings were cross‐checked by a *Lhukonzo* native speaker and a professional teacher against the English translation, for accuracy.
Generating initial codes	The translated data were organized (coded) systematically throughout the complete set of data.
Searching for themes	The codes were organized into potential themes by gathering all data relevant to each potential theme.
Reviewing themes	Checked if the themes worked regarding the coded extracts and the entire data set.
Defining and naming themes	Ongoing analysis from three independent researchers to refine the specifics of each theme, and the overall story the analysis tells, generating clear definitions and names for each theme.
Producing the report	Thereafter, a discussion was written for each theme relating to the analysis of the research question.

### 2.8 Ethical considerations

The study was performed following the ethical standards as laid down in the 1964 Declaration of Helsinki. Ethical approval was granted by The AIDS Support Organisation Research Ethical Committee (Reference number TASO‐2023‐252). Informed and signed consent were obtained individually from the lactating mothers attending the postnatal clinic of Bwera General Hospital, Kasese district, Western Uganda.

## 3 RESULTS

### 3.1 Background characteristics of study participants

A total of 94 eligible lactating mothers completed both the sensory evaluation study and FGDs. The mean age of the caregivers was 25.6 years. Only 36% of the lactating mothers had at least completed primary level education, while 37% and 63% of the mothers had infants, 1–5 and 6–23 months, respectively.

### 3.2 Sensory evaluation and acceptability of study composite dishes

The proportion of participants who scored the study composite dishes using a five‐point facial hedonic scale is shown in Table 3.

**TABLE 3:** The proportion of lactating mothers who scored the study composite dishes using a five‐point facial hedonic scale.

Composite dish	Attributes	Dislike very much *n* (%)	Dislike *n* (%)	Neither like nor dislike *n* (%)	Like much *n* (%)	Like very much *n* (%)
OFSP+IBCB	Color	0 (0)	0 (0)	1 (1.1)	15 (16)	78 (83)
	Texture	2 (2.1)	10 (10.6)	32 (34)	36 (38.3)	14 (14.9)
	Aroma	0 (0)	1 (1.1)	16 (17)	30 (31.9)	47 (50)
	Taste	1 (1.1)	4 (4.3)	3 (3.2)	31 (32.9)	55 (58.7)
	Overall acceptability	0 (0)	2 (2.1)	19 (20.2)	30 (31.9)	43 (45.7)
WFSP+NIBCB	Color	2 (2.1)	4 (4.3)	17 (18.5)	51 (54.3)	20 (21.3)
	Texture	0 (0)	4 (4.3)	24 (25.5)	46 (48.9)	20 (21.3)
	Aroma	2 (2.1)	4 (4.3)	12 (12.8)	53 (56.4)	23 (24.5)
	Taste	0 (0)	9 (9.6)	3 (3.2)	41 (43.6)	41 (43.6)
	Overall acceptability	1 (1.1)	12 (12.8)	10 (10.6)	44 (46.8)	26 (27.7)

Abbreviations: OFSP+IBCB, a combination of orange‐fleshed sweet potato and iron‐biofortified common bean; WFSP+NIBCB, a combination of white‐fleshed sweet potato and non‐iron biofortified common bean.

For the test composite dish (IBCB+OFSP), a high proportion of lactating mothers at 83%, 38.3%, 50%, 58.5%, and 45.7% gave a score of like very much (5), like much (4), like very much(5), like very much (5), like very much (5) for the color, texture, aroma, taste, and overall acceptability, respectively. Furthermore, in the control composite dish (NIBCB+WFSP), a high proportion of lactating mothers at 54.3%, 48.9%, 56.4%, and 46.4% gave a score of like much (4) for color, texture, aroma, and general acceptability, respectively. However, 41% scored taste either like much or like very much.

### 3.3 Sensory acceptability of the study composite dishes

A binary outcome for acceptability (yes or no) was created for each sensory attribute. An attribute was considered acceptable if the lactating mother scored it either 4 (like much) or 5 (like very much) on the five‐point hedonic facial scale. Table 4 shows the results from the chi‐square test for the association between each sensory attribute of the study composite foods and acceptability.

**TABLE 4:** Association between sensory attribute and acceptability of study composite foods.

Sensory attribute	Acceptability *N* = 94 across rows		Chi‐square	*p*‐value
	Yes, *n* (%)	No, *n* (%)		
Color				
OFSP+IBCB	93 (98.9)	1 (1.1)	21.8	<.0001
WFSP+NIBCB	72 (76.6)	22 (23.4)		
Texture				
OFSP+IBCB	50 (53.2)	44 (46.8)	5.78	.02
WFSP+NIBCB	66 (70.2)	28 (29.8)		
Aroma				
OFSP+IBCB	77 (81.9)	17 (18.1)	0.04	.851
WFSP+NIBCB	76 (80.9)	18 (19.1)		
Taste				
OFSP+IBCB	86 (91.5)	8 (8.5)	0.89	.344
WFSP+NIBCB	82 (87.2)	12 (12.8)		
General acceptability				
OFSP+IBCB	73 (77.7)	21 (22.3)	0.12	.73
WFSP+NIBCB	71 (75.5)	23 (24.5)		

Abbreviations: OFSP+IBCB, a combination of orange‐fleshed sweet potato and iron‐biofortified common bean; WFSP+NIBCB, a combination of white‐fleshed sweet potato and non‐iron biofortified common bean.

The color of OFSP+IBCB was more significantly accepted than that of WFSP+NIBCB (*p* < .0001). In contrast, the texture of WFSP+NIBCB was significantly more accepted than that of OFSP+IBCB (*p* = .02). However, there was no significant difference in acceptability between OFSP+IBCB for the other attributes including aroma (*p* = .851), taste (*p* = .344), and general acceptability (*p* = .73).

### 3.4 Perceptions on the consumption of study composite foods

Data saturation was achieved on the seventh FGD. Six key themes emerged reflecting issues important for the future consumption of OFSP+IBCB. These themes included sensory acceptability, feasibility to prepare, availability, affordability, sustainable supply, nutritional value, and health benefits of OFSP+IBCB.

#### 3.4.1 Sensory acceptability

The majority of lactating mothers reported no barriers to choosing OFSP+IBCB for sensory reasons. However, some mothers reported that they would find it difficult to choose OFSP because of its soft texture. Below are the different perceptions of lactating mothers based on the sensory attributes.

##### Color

The orange color of the OFSP, which was a component of the OFSP+IBCB dish, was attractive to the lactating mothers. They perceived that their children would also accept it if allowed to access it. Besides, they noted that the color of the IBCB and NIBCB in the OFSP+IBCB dish and WFSP+NIBCB dish was not different, respectively. However, they noticed a difference in the color of sweet potatoes in the two study dishes.

“That dish which had the yellow sweet potato was very attractive, it can stimulate appetite at first sight” (A mother of a 1‐month‐old baby)

“I did not notice any difference in the color of beans between the two dishes. However, I noticed the yellow color of the sweet potatoes in one dish and the white color of the sweet potatoes in the other dish. For sure this bright yellow color attracts you to eat this food. I like it” (A mother of a 3‐month‐old baby).

“…the beans used to prepare the two dishes are similar in color. It is the sweet potatoes that are different in color. Please health worker, tell us where that yellow sweet potato is accessible. I am sure my twins will like it because of that attractive bright yellow color” (A mother of 7‐month‐old twins).

“I would prefer OFSP to WFSP because the yellow color for OFSP is attractive to my child. I say this because my child likes to eat cooked pumpkin probably of its yellow color. Currently, my child eats the same food we prepare as a family. Therefore, I would buy the OFSP to prepare for all the household members including myself and my child to enjoy. For the common beans, they look the same, I would just buy whatever I find on the market” (A mother of a 16‐month‐old child).

##### Texture

The majority of lactating mothers complained of the soft texture in OFSP compared to the WFSP. However, they noted that this soft texture they observed in OFSP would be good for their children during complementary feeding. In contrast, they noted that the texture of IBCB and NIBCB was similar.

“… this OFSP is not bad. However, it has a very soft texture compared to our WFSP. If the plant breeders can work on improving the texture of OFSP, then it would be the best” (A mother of a 3‐month‐old infant).

“This OFSP is too soft compared to the WFSP. I guess this soft OFSP would be good for preparing complementary foods for our children” (A mother of a 9‐month‐old baby).

#### 3.4.2 Feasibility to prepare IBCB and OFSP

The mothers noted they would use the IBCB+OFSP if they cooked quickly, and they had adequate time, and skills to prepare them.

“…you see the problem with common beans, they take a long time to cook. You need to wait a little longer until they are ready. Sometimes you have a rush program, you can't wait for the beans that cook for over four hours. I will need to know whether the IBCB get cooked faster compared to our conventional beans” (A mother of a 6‐month‐old infant).

“Recently we have to share our time between cooking and our jobs. So, we need something which cooks quickly to leave at home for the household members, and for me to pack in my container for lunch while at my place of work. I would use the IBCB if they take a shorter period to cook compared to our conventional common bean. We no longer eat conventional common beans at home because it takes longer to cook. Sweet potato is fine it cooks faster either by boiling or steaming” (A mother of a 9‐month‐old baby).

“Common bean takes longer to cook compared to sweet potato, you waste a lot of time waiting for it to get ready. In the long run, you waste a lot of fuel on it. On average, how many hours does that IBCB variety take to get ready if you cook it on a full medium charcoal stove?” (A mother of a 10 month old baby)

Some mothers wondered whether the cooking methods for IBCB and OFSP are similar to those used to cook NIBCB and WFSP.

“Are the IBCB and OFSP require different cooking methods and skills to prepare them for eating or they are cooked the same way as our locally available WFSP and NIBCB? Please let us know” (A teenage mother of a 6 month old infant).

“…I am pretty sure that the local methods we use to cook WFSP and NIBCB are not any different from those we have to use while cooking OFSP and IBCB, respectively” (A mother of a 13‐month‐old child)

“Common bean remains common bean irrespective of variety. We usually cook by boiling it with either prior soaking or no prior soaking. Even you can remove the seed coat before boiling. The same way with sweet potatoes, whether WFSP or OFSP they are all sweet potatoes. The commonly used methods to cook sweet potatoes are either boiling or steaming”

(A mother of an 11‐month‐old infant).

#### 3.4.3 Availability of IBCB and OFSP

The majority of mothers showed interest in knowing the market where they could find the OFSP and IBCB.

“…tell us where that yellow sweet potato can be found to buy it. I am sure my twins will like it because of that attractive bright yellow color” (A mother of 7‐month‐old twins).

“Where would one find the vines for the OFSP and seeds for IBCB if one wants to plant them? This would help me grow them myself instead of buying them from the market” (A mother of a 10‐month‐old infant).

#### 3.4.4 Affordability of IBCB and OFSP

The lactating mothers expressed the issue of affordability of IBCB and OFSP and the majority noted that they would consume them if they were cheaper to purchase from the market, produce in the gardens, and cook before eating.

“How much is the cost of IBCB and OFSP each? Is it cheaper than our locally available NIBCB and WFSP? I would buy for household consumption if it is offered at an affordable price” (A mother of a 10‐month‐old infant)

“Cooking common bean is very expensive because it consumes a lot of fuel. Common beans take a long time to cook hence they consume a lot of fuel such as charcoal and firewood which we usually use for cooking here. We need common bean which cooks fast to save fuel” (A mother of an 11‐month‐old infant).

“How much could the vines and seeds for OFSP and IBCB cost, respectively if someone wanted to plant them? You see for us we are used to plant WFSP and NIBCB. I have picked interest in growing both IBCB and OFSP, but where can I access the seeds and vines, respectively for planting? I need to give them a try” (A mother of an 18‐month‐old child).

“…growing would be good, but it might be expensive in the long run if those IBCB and OFSP are easily attacked by pests and diseases or are not drought resistant” (A mother to a 6‐month‐old infant)

“…those newly introduced genetically modified biofortified foods are not like our local breeds. They are easily attacked by pests and diseases. So, it means that you will have to buy pesticides. That means incurring more costs. Our conventional NIBCB and WFSP are usually grown minus applying pesticides. Are these IBCB and OFSP pest resistant?” (A mother of a 21‐month‐old child).

#### 3.4.5 Sustainable supply of IBCB and NIBCB

The majority of lactating mothers raised a concern about a sustainable supply of the IBCB and OFSP in the food supply chain.

“…I would consume the OFSP and IBCB if I could find it on the market always for me to buy. The challenge is to go to the market and you don't find it whenever you want it” (A mother of a 16‐month‐old child).

“You see some time back (2 years back), the Ministry of Agriculture officials introduced some common beans in our community. They told us they are nutritious and give a high yield. However, I was demoralized when I looked for seeds on the market for three consecutive seasons to plant them, and I could not find them. How I wish you let us know where we could find these seeds for OFSP and IBCB so that we can plant them season after season”

“…Some of us are working mothers, we usually eat away from home in places such as restaurants. I wonder whether the restaurant people can cook OFSP and IBCB as they do for NIBCB and WFSP so that people who eat away from home can consume the OFSP and IBCB from such eating outlets” (A mother of a 22‐month‐old baby)

#### 3.4.6 Nutritional value and health benefits

The majority of mothers suggested that they would buy and consume OFSP if they knew that their nutritional value and health benefits were superior to that of WFSP. However, they noted that the nutritional value and health benefits of IBCB and NIBCB were similar. Either the former or the latter was noted by at least one lactating mother in each of the seven FGDs conducted.

“You see OFSP is very soft with many threadlike fibers compared to WFSP. For sure it isn't appetizing to eat OFSP. I will only choose it if I am sure that it is more nutritious than WFSP” (A mother to an 11‐month‐old infant).

“…I will only consider eating OFSP combined with either IBCB or NIBCB if I know that OFSP has more health benefits than WFSP. My reason is that OFSP is too soft compared to WFSP. However, IBCB and NIBCB are very similar in every sensory attribute and nutritional value. So, I can choose any available to eat as a soup with OFSP” (A mother to a 9‐month‐old infant).

“…I meant that IBCB and NIBCB appear similar in everything such as color, shape, size, and taste. Because of this similarity, nutritional value is also the same. So, I would choose any of the common bean varieties I find on the market” (A mother to a 9‐month‐old infant).

“…yes, I think OFSP has nutrients that have health benefits such as good eyesight because of its yellowish color similar to carrots. During the immunization visits of my firstborn, health workers taught us that foods with yellow color such as carrots are good for eyesight. I would also consume OFSP probably because of its yellow color and the associated health benefits for eyesight” (A mother to a 3‐month‐old infant).

## 4 DISCUSSION

This study explored lactating mothers' perceptions and sensory acceptability of a PVA carotenoid–iron‐rich composite dish prepared from a combination of OFSP and IBCB. A combination of WFSP and conventional NIBCB is commonly consumed in Uganda. However, this combination is low in PVA carotenoids and iron. The retinol activity equivalent (RAE) + iron composition in OFSP+ IBCB (test food) and WFSP+NIBCB (control food) used in this study was 3088 and 711 μgRAE+5.1 mg/100 g, respectively (Table 1). The RDA for iron and retinol for lactating women is 9.5 mg/day and 1300 μgRAE, respectively (Gannon et al., 2020; Kominiarek & Rajan, 2016; Ross et al., 2014). Therefore, approximately 100 g of IBCB and only 42 g from OFSP+IBCB would be needed to meet the RDA requirements for iron and retinol required for lactating mothers. In contrast, nearly 200 g of NIBCB and 200 g of WFSP from WFSP+NIBCB would be needed to meet the RDA requirements for iron and retinol required for lactating mothers. This suggests that compared to the WFSP+NIBCB, a smaller portion of OFSP+IBCB is needed to meet RDA requirements for iron and retinol for lactating mothers.

To our knowledge, this is the first attempt to understand the maternal acceptability of homemade composite dishes prepared from foods biofortified with PVA carotenoid and iron, versus the low iron‐PVAC dishes prepared from conventional foods in Uganda. In the present study, the color of the IBCB+ OFSP dish was highly significantly accepted compared to that of the NIBCB+ OFSP dish, probably because of the deep orange color of the OFSP, which was attractive to the lactating mothers as they reported in the FGDs. In contrast, the lactating mothers seemed not to recognize any color difference between IBCB and NIBCB, as they emphasized during FGDs that every sensory attribute was similar between IBCB and NIBCB. Therefore, the color acceptability of OFSP+IBCB could have between attributed to the color of OFSP, but not IBCB. This finding is similar to the previous studies which reported that PVA‐biofortified foods such as OFSP and either yellow maize or cassava were acceptable to the potential consumers because of the attractive deep orange color in the former and yellow color in the latter (Amod et al., 2016; Govender et al., 2014, 2019a; Talsma et al., 2013). It is worth noting that the orange and yellow colors observed in PVA‐biofortified foods including OFSP are due to the increased PVA carotenoids added to these staples during biofortification (Yuan et al., 2015). Findings from this present study confirm that the yellow or orange color observed in PVA‐biofortified foods improves the acceptability of other foods if they are combined with other foods with less attractive colors. In South Africa, Govender and colleagues demonstrated that because of the appealing color of OFSP and yellow PVA biofortified maize, consumers were accepting and had positive perceptions of non‐biofortified foods, if they were combined with either OFSP or PVA‐biofortified maize (Govender et al., 2019a). However, it is worth noting that the present study used a test composite dish prepared from a combination of biofortified foods including OFSP and IBCB.

During FGDs, mothers raised a suggestion of the likelihood of their children's acceptability of the complementary food prepared from the common bean and OFSP because of the attractive color they observed in OFSP. Such suggestions from lactating mothers confirm that lactating mothers are gatekeepers of complementary foods (CFs), they feed to their children (Buzigi et al., 2020a; Food and Agriculture of the United Nations, 2017). Moreover, previous studies have shown that, based on the appealing yellow or orange color observed in PVA biofortified foods, caregivers have shown interest in preparing CFs using PVA‐biofortified foods (Amod et al., 2016; Govender et al., 2014). The lactating mothers' suggestions may also indicate the necessity of developing an innovative complementary food blend rich in PVA carotenoids and iron from OFSP+IBCB and testing its acceptability among their children. The lactating mothers' suggestion is plausible because 6‐ to 23‐month‐old children in low‐income countries such as Uganda are at a higher risk of developing ID and VAD since they are predominantly fed CFs that are low in iron and vitamin A (Amaral et al., 2018; Ekesa et al., 2019; Uganda Bureau of Statistics & Inner City Fund, 2018). Moreover, developing and testing child acceptability of PVA carotenoid–iron‐rich CFs and testing its acceptability among children in the age range of complementary feeding is feasible (Buzigi et al., 2020b).

The texture of IBCB+OFSP was not acceptable to the lactating mothers compared to that of NIBCB and WFSP (*p* = .02). The unacceptability of the IBCB+OFSP dish was linked to the soft texture of the OFSP but not IBCB, as study participants indicated during the FGDs that the sensory properties of IBCB and NIBCB were similar. This finding is not surprising because the majority of OFSP varieties released in low‐ and middle‐income countries including Uganda have high moisture content, compared to the WFSP (Alam et al., 2016; Neela & Fanta, 2019), hence their softness. The softness reported by lactating mothers in the present study indicates the necessity of increasing the dry matter content of OFSP. Moreover, it is feasible to increase the dry matter content of OFSP (Ginting et al., 2017). In contrast, during the FGDs, lactating mothers used the softness of OFSP as a justification for the potential use of OFSP as a complementary food for their children. This justification is plausible because it is recommended that CFs should be soft to prevent choking in children (Food and Agriculture of the United Nations, 2017).

Aroma, taste, and general acceptability were scored highly by the lactating mothers and not significantly different between OFSP+IBCB and WFSP+NIBCB. Such findings indicate the potential of replacing conventional WFSP+NIBCB with OFSP+IBCB. Similar findings have been reported in South Africa (Govender et al., 2019a). However, the South African study did not have a composite dish having a combination of IBCB and OFSP as it only focused on the traditional composite dishes prepared with either OFSP or PVA‐biofortified maize (Govender et al., 2019a). Moreover, the South African study recruited adult males and females (Govender et al., 2019a), compared to this present study, which recruited lactating mothers, a target group with high physiological demands for iron and vitamin A (Gannon et al., 2020; Khayat et al., 2017).

During FGDs, some mothers showed interest in knowing where they would access the vines and seeds for OFSP and IBCB, respectively, so that they could grow them for their home consumption, and even sell the excess to improve their incomes. These findings indicate the possibility of adopting the use of study biofortified foods in the study participants. Similar findings of the potential to adopt the growing OFSP have been reported elsewhere in Eastern Uganda (Ssebuliba et al., 2006). Moreover, the adoption of IBCB has the potential to improve household consumption of IBCB (Vaiknoras & Larochelle, 2021).

Considering the feasibility of preparing OFSP and IBCB, mothers were worried about the long cooking time for common beans and the high fuel costs associated with cooking common beans. This finding is consistent with previous studies conducted in Uganda, which showed that consumers were afraid to use common beans because of their long cooking time and high fuel consumption (Buzigi et al., 2020a), or they preferred common bean processing methods that reduce the cooking time of common bean and hence save fuel (Aseete et al., 2018). It is worth noting that the cooking time for NIBCB and IBCB used in this present study during the preparation of the study composite dishes was approximately 1.5 and 3.5 h, respectively. Such a high cooking time observed for IBCB compared to NIBCB would prevent the lactating mothers from cooking IBCB for household consumption. However, the peer mothers who prepared the study composite dishes first soaked common beans before cooking them. This was plausible because cooking IBCB with prior soaking may reduce the cooking time of common beans (Muroki et al., 2023).

During FGDs, lactating mothers indicated that they would consume OFSP+IBCB if they knew that they were nutritious or if eating them would provide any health benefits. For example, one study participant emphasized that she would consume a combination of OFSP and IBCB because she knew from a previous nutrition education program, that the OFSP provided health benefits to the eye. This finding informs that there is a need to promote the consumption of biofortified foods such as OFSP and IBCB through community nutrition education. The education messages should focus on the nutritional value of biofortified foods and the health benefits of their consumption. It is worth noting that previous studies have shown that the sensory acceptability and willingness to pay for biofortified foods may be improved through nutrition and health promotion program activities such as providing information, education, and communication about the nutritional and health benefits of eating biofortified food crops such as biofortified yellow cassava and IBCB (Bechoff et al., 2018; Oparinde, Banerji, et al., 2016; Oparinde, Birol, et al., 2016). For example, in Rwanda, the sensory acceptability of IBCB was higher among consumers who had prior sensitization and awareness about the nutritional benefits of consuming IBCB (Murekezi et al., 2017).

## 5 CONCLUSIONS

The PVA carotenoid‐rich sweet potato, OFSP served with a high iron common bean, IBCB (OFSP+IBCB) was significantly accepted for color, but not texture, compared to the low PVA conventional sweet potato, WFSP served with the low iron conventional common bean, NIBCB (WFSP+NIBCB). However, for overall acceptability, OFSP+IBCB and WFSP+ NIBCB were equally acceptable, suggesting that the biofortified, OFSP+IBCB dish has the potential to replace the staple non‐biofortified, WFSP+NIBCB dish among lactating mothers in the study area. Lactating mothers had positive perceptions of using OFSP and IBCB if they could cook fast, and provide health benefits upon consumption, with a stable availability and economic access in the food supply chain.

## AUTHOR CONTRIBUTIONS

**Edward Buzigi:** Conceptualization (lead); data curation (lead); formal analysis (lead); investigation (lead); methodology (lead); project administration (equal); resources (equal); software (equal); supervision (equal); validation (equal); visualization (equal); writing – original draft (lead); writing – review and editing (lead). **Kirthee Pillay:** Conceptualization (equal); investigation (equal); methodology (equal); supervision (equal); validation (equal); visualization (equal); writing – review and editing (equal). **Muthulisi Siwela:** Conceptualization (equal); data curation (equal); methodology (equal); resources (equal); supervision (equal); writing – review and editing (equal). **Blessing Mkhwanazi:** Conceptualization (equal); funding acquisition (equal); methodology (equal); validation (equal); visualization (equal); writing – review and editing (equal). **Mjabuliseni Ngidi:** Conceptualization (equal); funding acquisition (equal); methodology (equal); supervision (equal); visualization (equal); writing – review and editing (equal). **Babra Muhindo Mahinda:** Conceptualization (equal); investigation (equal); methodology (equal); resources (equal); visualization (equal); writing – review and editing (equal). **Joshua Ssozi:** Conceptualization (equal); methodology (equal); writing – review and editing (equal). **Evyline Barugahara Isingoma:** Methodology (equal); writing – review and editing (equal).

## FUNDING INFORMATION

This research was funded through the postdoctoral scholarship awarded to Edward Buzigi in the School of Agricultural, Earth, and Environmental Science within the College of Agriculture, Engineering, and Science at the University of Kwa‐Zulu Natal, South Africa.

## CONFLICT OF INTEREST STATEMENT

The authors declare that they have no competing interests.

## ETHICS APPROVAL AND CONSENT TO PARTICIPATE

The study was performed following the ethical standards as laid down in the 1964 Declaration of Helsinki. Ethical approval was granted by the Research Ethical Committee at The AIDS Support Organisation Research Ethical Committee (Reference number TASO‐2023‐252). Informed and signed consent were obtained individually from the mothers in the postnatal clinicians who participated in the study. Informed consent was taken from legally authorized representatives and/or guardians of all participants who were below 18 years old and those without formal education.

## Data Availability

The datasets used and/or analyzed in the present study are available from the corresponding author on reasonable request.
